# The genome sequence of the Fruity Milkcap, *Lactarius evosmus *(Kühner & Romagn., 1954)

**DOI:** 10.12688/wellcomeopenres.19910.1

**Published:** 2023-10-18

**Authors:** Penny Cullington, Brian Douglas, Kieran Woof, Ester Gaya, Royal Botanic Gardens Kew Genome Acquisition Lab, Darwin Tree of Life Barcoding collective, Wellcome Sanger Institute Tree of Life programme, Wellcome Sanger Institute Scientific Operations: DNA Pipelines collective, Tree of Life Core Informatics collective, Darwin Tree of Life Consortium

**Affiliations:** 1 British Mycological Society, London, England, UK; 2 Royal Botanic Gardens Kew, Richmond, England, UK

**Keywords:** Lactarius evosmus, Fruity Milkcap, genome sequence, chromosomal, Russulales

## Abstract

We present a genome assembly from an individual *Lactarius evosmus* (Fruity Milkcap; Basidiomycota; Agaricomycetes; Russulales; Russulaceae). The genome sequence is 57.2 megabases in span. Most of the assembly is scaffolded into 11 chromosomal pseudomolecules. The mitochondrial genome has also been assembled and is 61.51 kilobases in length.

## Species taxonomy

Eukaryota; Fungi; Dikarya; Basidiomycota; Agaricomycotina; Agaricomycetes; Agaricomycetes incertae sedis; Russulales; Russulaceae; *Lactarius*; *Lactarius evosmus* (Kühner & Romagn., 1954) (NCBI:txid326039).

## Background

*Lactarius evosmus* (Fruity Milkcap) is a member of a large genus of mycorrhizal mushrooms well represented in the UK and widespread in Europe. The order Russulales comprises *Lactarius* and *Russula* – both gilled genera – together with some bracket, resupinate, and toothed fungi, and even some truffles. All members of this order of fungi have spores with strongly amyloid ornamentation (turning blue-black in Melzer’s reagent). The genus *Lactarius* has one unique feature: damage to the gills promptly produces the tell-tale ‘milk’ (latex), hence its common English name, Milkcap. The latex – white or occasionally colourless when first exposed to air – often changes colour to yellow, pink, greenish-grey, or even violet-purple, depending on the species. Ecologically, some *Lactarius* species associate with deciduous trees, others with conifers, some are host specific, and still others are generalists ( Kibby, 2020).

*Lactarius evosmus* is ectomycorrhizal with *Quercus*, *Populus*, and *Salix* species, favouring calcareous soils, although it is not one of the UK’s more common Milkcap species. Since the late 1990s, we have become aware of it fruiting prolifically in association with *Helianthemum nummularium* (Rock Rose) in open chalk downland with no trees present – one of very few members of this genus to do this – though there are growing numbers of other mycorrhizal genera well represented in such habitats. The specimen used for genome sequencing was collected from Watlington Hill on the Chiltern escarpment in Oxfordshire in association with *Helianthemum*. The species can regularly be seen here in extraordinary numbers during the autumn months together with a range of other mushroom species.

**Figure 1.**
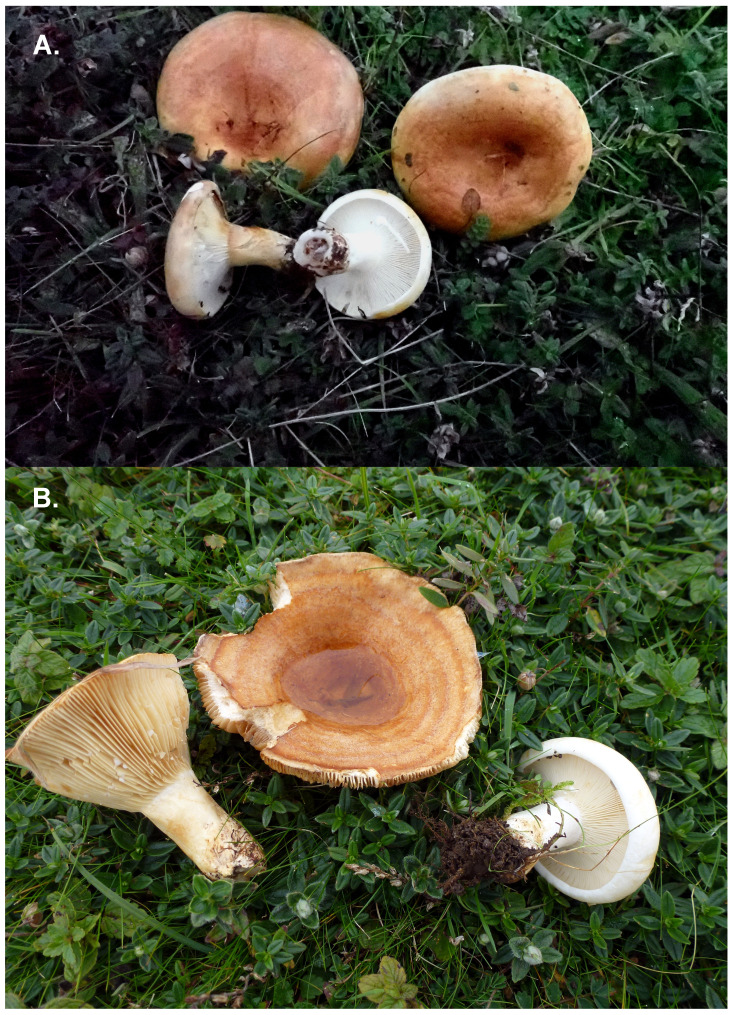
Photograph of the *Lactarius evosmus* (gfLacEvos1) specimen used for genome sequencing.

*Lactarius evosmus* is a medium to large Milkcap ( Figure 1). The cap is 6–10(15) cm across, tightly inrolled at first, then becoming less so as it expands with a sunken centre, surface ± smooth, sticky in damp conditions, cream to yellowish clay-buff and developing distinct radial darker zones more ochre-brown as it matures. The gills are fairly crowded, adnate to slightly decurrent or with decurrent tooth; at first cream, later pinkish buff. The latex is abundant, remaining white as it dries, with very acrid taste. Stem to 6 × 3 cm in dimension, dry, firm and smooth, pale cream, becoming more ochre with age. The smell is significantly fruity, reminiscent of apples and similar to that in *Russula fellea* – hence its common species name. The sporeprint is pinkish-buff. The basidia are 4-spored. The spores average 8 × 6.5 µm, ellipsoid, and are ornamented with low warts and ridges, which are amyloid. Cystidia are present on the gill edge but are not considered significant for species identification ( Noordeloos *et al.*, 2018).

The species is very similar in appearance to both *L. acerrimus* and *L. zonarius*. The first, however, has 2-spored basidia; the second has a notably pubescent margin in early stages and a stem with small ‘pock-marks’. All three species can occur under *Quercus*, but only *L. evosmus* has the distinct apple smell. To date neither *L. acerrimus* nor *L. zonarius* occur with *Helianthemum*.

The genome of *Lactarius evosmus* was sequenced as part of the Darwin Tree of Life Project, a collaborative effort to sequence all named eukaryotic species in the Atlantic Archipelago of Britain and Ireland. Here we present a complete chromosomal genome sequence for a *Lactarius evosmus* specimen from Watlington Hill, found in association with *Helianthemum nummularium*.

## Genome sequence report

The genome was sequenced from a specimen of *Lactarius evosmus* collected from Watlington Hill, Oxfordshire (51.64, –0.99). A total of 326-fold coverage in Pacific Biosciences single-molecule HiFi long reads was generated. Primary assembly contigs were scaffolded with chromosome conformation Hi-C data. Manual assembly curation corrected three missing joins or misjoins and removed one haplotypic duplication, reducing the assembly length by 22.58% and the scaffold number by 98.18%, and increasing the scaffold N50 by 10.85%.

**Figure 2.**
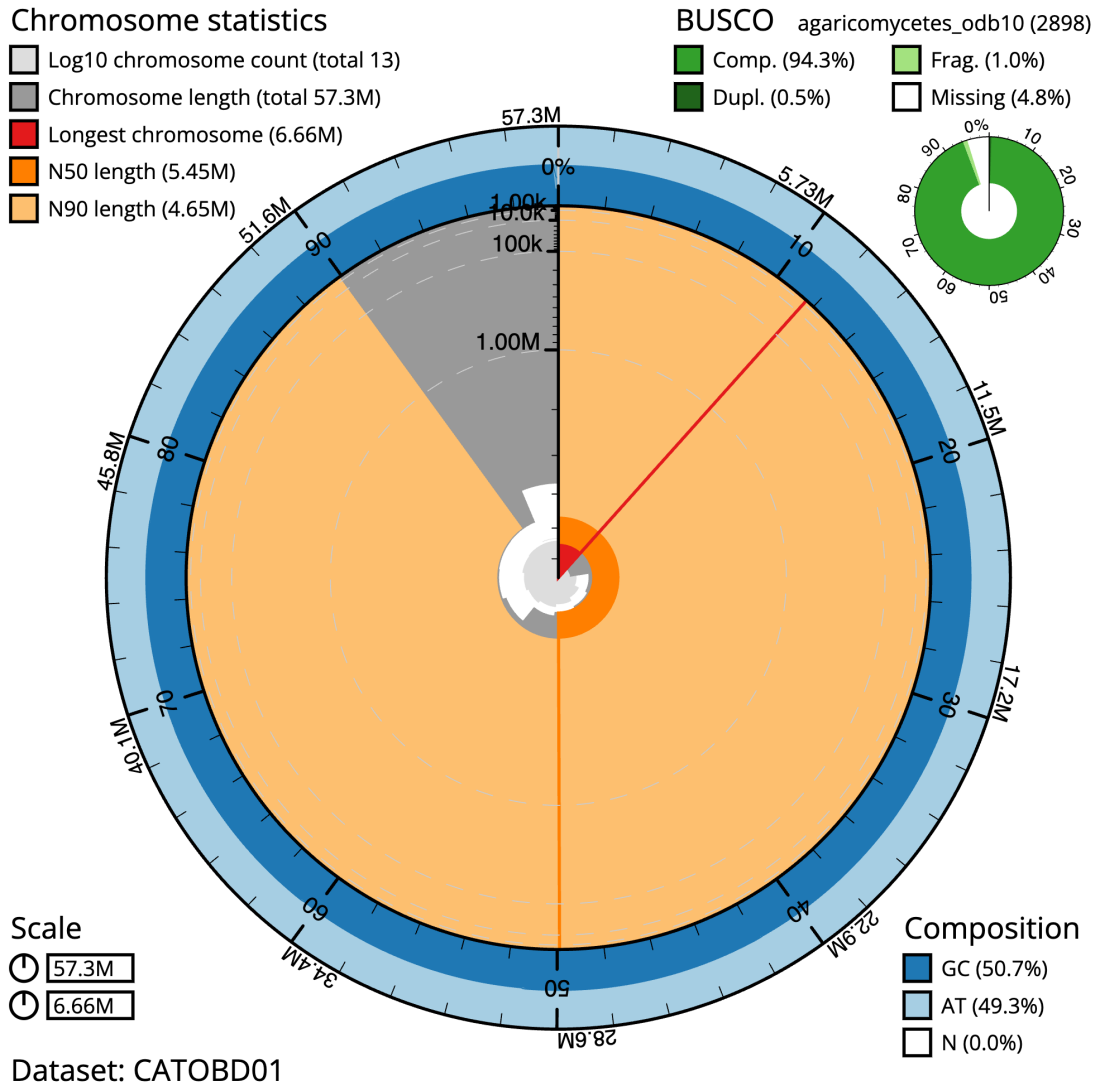
Genome assembly of *Lactarius evosmus*, gfLacEvos1.1: metrics. The BlobToolKit Snailplot shows N50 metrics and BUSCO gene completeness. The main plot is divided into 1,000 size-ordered bins around the circumference with each bin representing 0.1% of the 57,293,171 bp assembly. The distribution of scaffold lengths is shown in dark grey with the plot radius scaled to the longest scaffold present in the assembly (6,657,085 bp, shown in red). Orange and pale-orange arcs show the N50 and N90 scaffold lengths (5,448,807 and 4,650,992 bp), respectively. The pale grey spiral shows the cumulative scaffold count on a log scale with white scale lines showing successive orders of magnitude. The blue and pale-blue area around the outside of the plot shows the distribution of GC, AT and N percentages in the same bins as the inner plot. A summary of complete, fragmented, duplicated and missing BUSCO genes in the agaricomycetes_odb10 set is shown in the top right. An interactive version of this figure is available at https://blobtoolkit.genomehubs.org/view/gfLacEvos1.1/dataset/CATOBD01/snail.

The final assembly has a total length of 57.2 Mb in 12 sequence scaffolds with a scaffold N50 of 5.4 Mb ( Table 1). Most (99.89%) of the assembly sequence was assigned to 11 chromosomal-level scaffolds. Chromosome-scale scaffolds confirmed by the Hi-C data are named in order of size ( Figure 2– Figure 5; Table 2). While not fully phased, the assembly deposited is of one haplotype. Contigs corresponding to the second haplotype have also been deposited. The mitochondrial genome was also assembled and can be found as a contig within the multifasta file of the genome submission.

**Table 1.** Genome data for *Lactarius evosmus*, gfLacEvos1.1.

Project accession data		
Assembly identifier	gfLacEvos1.1	
Species	*Lactarius evosmus*	
Specimen	gfLacEvos1	
NCBI taxonomy ID	326039	
BioProject	PRJEB58661	
BioSample ID	SAMEA110449669	
Isolate information	gfLacEvos1: cap (DNA sequencing, Hi-C scaffolding, RNA sequencing)	
Assembly metrics *		*Benchmark*
Consensus quality (QV)	78	*≥ 50*
*k*-mer completeness	100%	*≥ 95%*
BUSCO **	C:94.3%[S:93.8%,D:0.5%], F:1.0%,M:4.8%,n:2,898	*C ≥ 95%*
Percentage of assembly mapped to chromosomes	99.89%	*≥ 95%*
Sex chromosomes	-	*localised homologous * *pairs*
Organelles	Mitochondrial genome assembled	*complete single alleles*
Raw data accessions		
PacificBiosciences SEQUEL II	ERR10753936	
Hi-C Illumina	ERR10742411	
PolyA RNA-Seq Illumina	ERR11641118	
Genome assembly		
Assembly accession	GCA_951394315.1	
*Accession of alternate haplotype*	GCA_951394305.1	
Span (Mb)	57.2	
Number of contigs	24	
Contig N50 length (Mb)	4.1	
Number of scaffolds	12	
Scaffold N50 length (Mb)	5.4	
Longest scaffold (Mb)	6.7	

* Assembly metric benchmarks are adapted from column VGP-2020 of “Table 1: Proposed standards and metrics for defining genome assembly quality” from ( Rhie *et al.*, 2021). ** BUSCO scores based on the agaricomycetes_odb10 BUSCO set using v5.3.2. C = complete [S = single copy, D = duplicated], F = fragmented, M = missing, n = number of orthologues in comparison. A full set of BUSCO scores is available at https://blobtoolkit.genomehubs.org/view/gfLacEvos1.1/dataset/CATOBD01/busco.

**Figure 3.**
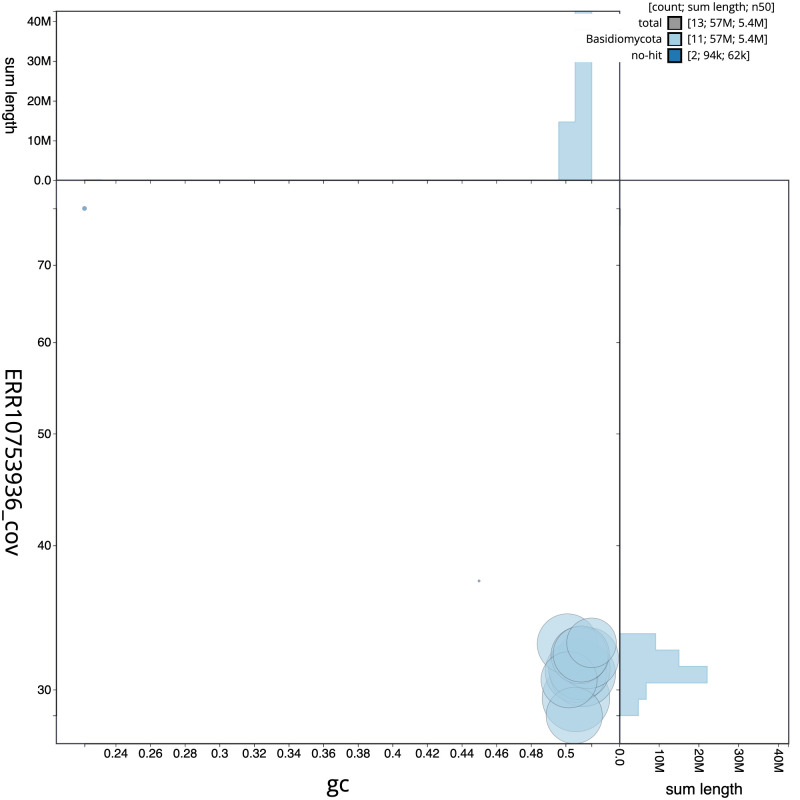
Genome assembly of *Lactarius evosmus*, gfLacEvos1.1: BlobToolKit GC-coverage plot. Scaffolds are coloured by phylum. Circles are sized in proportion to scaffold length. Histograms show the distribution of scaffold length sum along each axis. An interactive version of this figure is available at https://blobtoolkit.genomehubs.org/view/gfLacEvos1.1/dataset/CATOBD01/blob.

**Figure 4.**
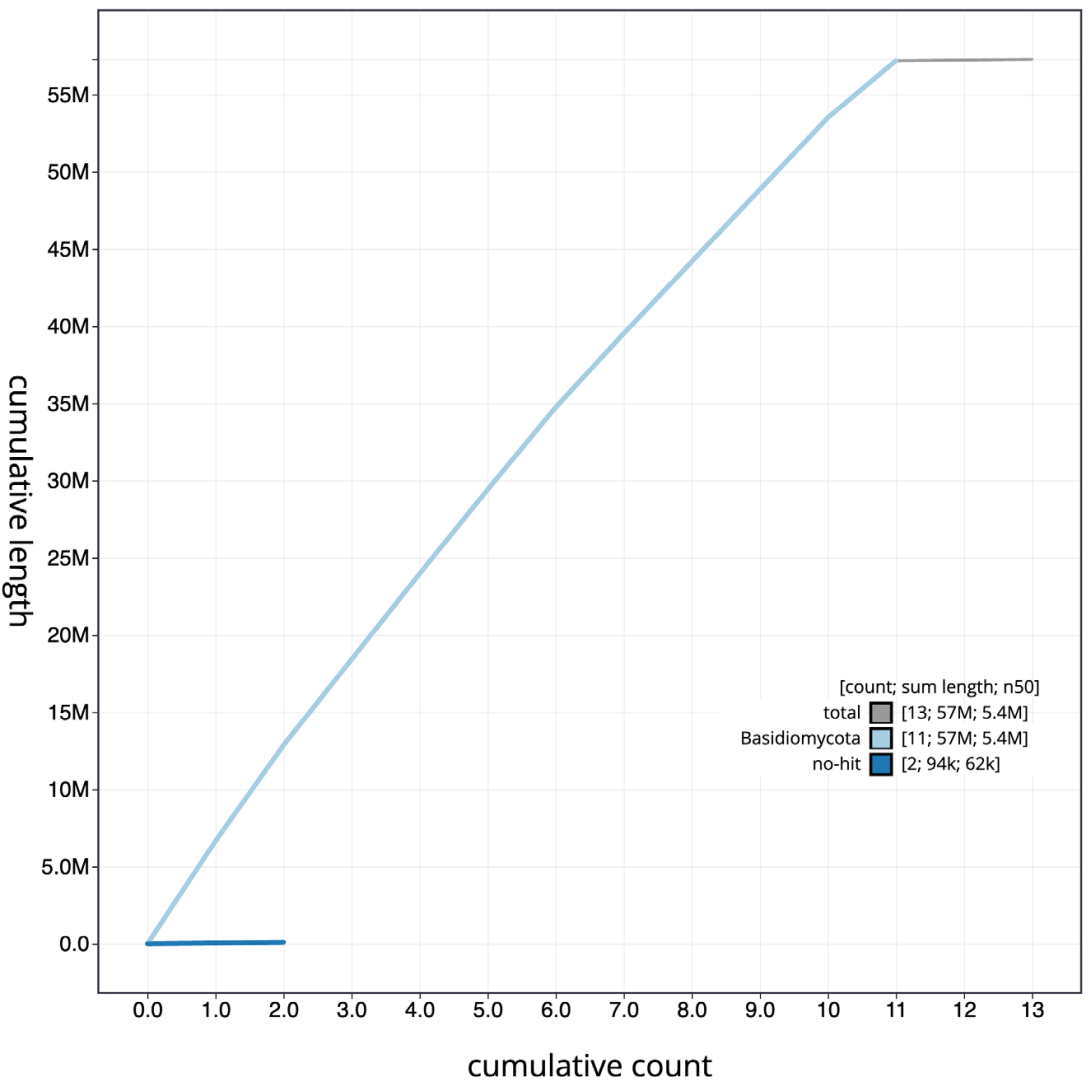
Genome assembly of *Lactarius evosmus*, gfLacEvos1.1: BlobToolKit cumulative sequence plot. The grey line shows cumulative length for all scaffolds. Coloured lines show cumulative lengths of scaffolds assigned to each phylum using the buscogenes taxrule. An interactive version of this figure is available at https://blobtoolkit.genomehubs.org/view/gfLacEvos1.1/dataset/CATOBD01/cumulative.

**Figure 5.**
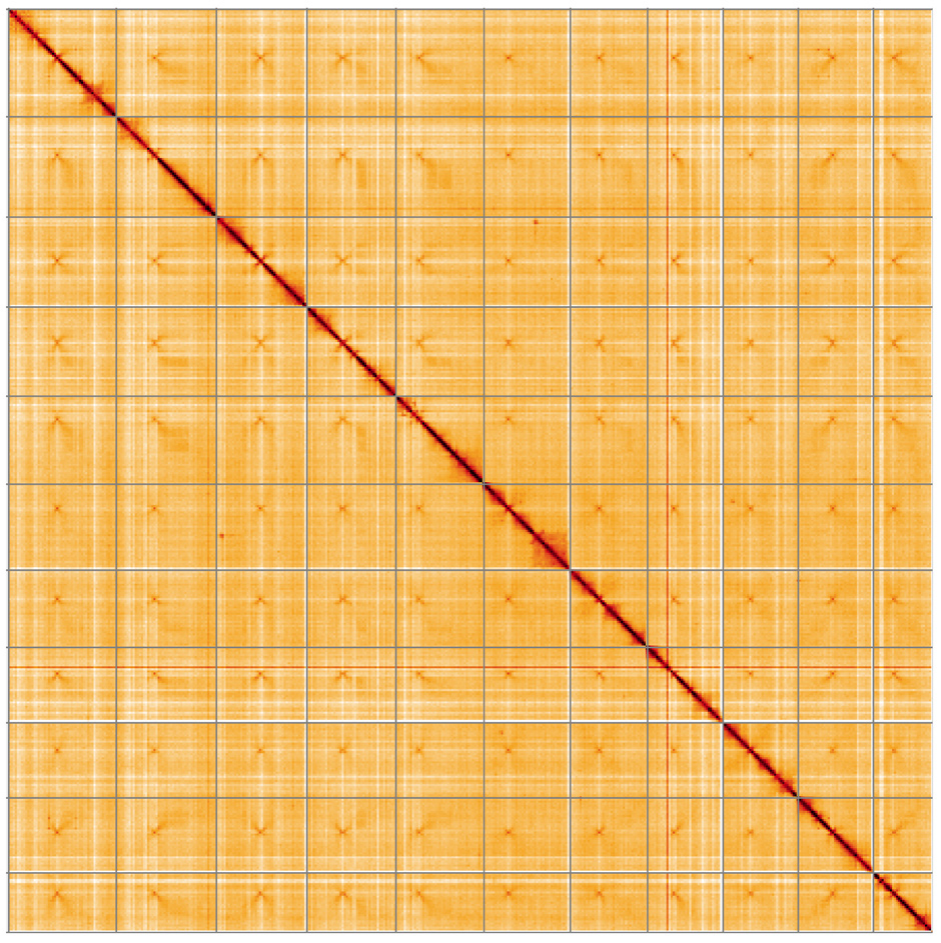
Genome assembly of *Lactarius evosmus*, gfLacEvos1.1: Hi-C contact map of the gfLacEvos1.1 assembly, visualised using HiGlass. Chromosomes are shown in order of size from left to right and top to bottom. An interactive version of this figure may be viewed at https://genome-note-higlass.tol.sanger.ac.uk/l/?d=a7wK8basTWa4jP_ndUAizg.

**Table 2.** Chromosomal pseudomolecules in the genome assembly of *Lactarius evosmus*, gfLacEvos1.

INSDC accession	Chromosome	Length (Mb)	GC%
OX596124.1	1	6.66	50.5
OX596125.1	2	6.21	51.0
OX596126.1	3	5.59	50.5
OX596127.1	4	5.52	51.0
OX596128.1	5	5.45	51.5
OX596129.1	6	5.33	50.0
OX596130.1	7	4.78	51.0
OX596131.1	8	4.67	50.5
OX596132.1	9	4.66	50.0
OX596133.1	10	4.65	51.0
OX596134.1	11	3.69	51.5
OX596135.1	MT	0.06	22.5

The estimated Quality Value (QV) of the final assembly is 78 with *k*-mer completeness of 100%, and the assembly has a BUSCO v5.3.2 completeness of 94.3% (single = 93.8%, duplicated = 0.5%), using the agaricomycetes_odb10 reference set ( *n* = 2,898).

Metadata for specimens, spectral estimates, sequencing runs, contaminants and pre-curation assembly statistics can be found at https://links.tol.sanger.ac.uk/species/326039.

## Methods

### Sample acquisition and nucleic acid extraction

A *Lactarius evosmus* (specimen ID KDTOL00275, ToLID gfLacEvos1) was collected from Watlington Hill, Oxfordshire, UK (latitude 51.64, longitude –0.99) on 2021-11-02. The specimen was handpicked from an area with *Helianthemum* sp. by Penny Cullington and transported to the Royal Botanic Gardens, Kew for snap freezing. The specimen was identified by Penny Cullington (British Mycological Society), confirmed by Brian Douglas (Royal Botanic Gardens Kew) and processed and snap-frozen in liquid nitrogen by Kieran Woof.

DNA was extracted at the Tree of Life laboratory, Wellcome Sanger Institute (WSI). The gfLacEvos1 sample was weighed and dissected on dry ice with tissue set aside for Hi-C sequencing. Tissue from the cap was cryogenically disrupted to a fine powder using a Covaris cryoPREP Automated Dry Pulveriser, receiving multiple impacts. High molecular weight (HMW) DNA was extracted using the Qiagen Plant MagAttract v2 DNA extraction kit. HMW DNA was sheared into an average fragment size of 12–20 kb in a Megaruptor 3 system with speed setting 30. Sheared DNA was purified by solid-phase reversible immobilisation using AMPure PB beads with a 1.8X ratio of beads to sample to remove the shorter fragments and concentrate the DNA sample. The concentration of the sheared and purified DNA was assessed using a Nanodrop spectrophotometer and Qubit Fluorometer and Qubit dsDNA High Sensitivity Assay kit. Fragment size distribution was evaluated by running the sample on the FemtoPulse system.

RNA was extracted from cap tissue of gfLacEvos1 in the Tree of Life Laboratory at the WSI using TRIzol, according to the manufacturer’s instructions. RNA was then eluted in 50 μl RNAse-free water and its concentration assessed using a Nanodrop spectrophotometer and Qubit Fluorometer using the Qubit RNA Broad-Range (BR) Assay kit. Analysis of the integrity of the RNA was done using Agilent RNA 6000 Pico Kit and Eukaryotic Total RNA assay.

### Sequencing

Pacific Biosciences HiFi circular consensus DNA sequencing libraries were constructed according to the manufacturers’ instructions. Poly(A) RNA-Seq libraries were constructed using the NEB Ultra II RNA Library Prep kit. DNA and RNA sequencing was performed by the Scientific Operations core at the WSI on Pacific Biosciences SEQUEL II (HiFi) and Illumina NovaSeq 6000 (RNA-Seq) instruments. Hi-C data were also generated from cap tissue of gfLacEvos1 using the Arima2 kit and sequenced on the Illumina NovaSeq 6000 instrument.

### Genome assembly, curation and evaluation

Assembly was carried out with Hifiasm ( Cheng *et al.*, 2021) and haplotypic duplication was identified and removed with purge_dups ( Guan *et al.*, 2020). The assembly was then scaffolded with Hi-C data ( Rao *et al.*, 2014) using YaHS ( Zhou *et al.*, 2023). The assembly was checked for contamination and corrected as described previously ( Howe *et al.*, 2021). Manual curation was performed using HiGlass ( Kerpedjiev *et al.*, 2018) and Pretext ( Harry, 2022). The mitochondrial genome was assembled using MitoHiFi ( Uliano-Silva *et al.*, 2023), which runs MitoFinder ( Allio *et al.*, 2020) or MITOS ( Bernt *et al.*, 2013) and uses these annotations to select the final mitochondrial contig and to ensure the general quality of the sequence.

A Hi-C map for the final assembly was produced using bwa-mem2 ( Vasimuddin *et al.*, 2019) in the Cooler file format ( Abdennur & Mirny, 2020). To assess the assembly metrics, the *k*-mer completeness and QV consensus quality values were calculated in Merqury ( Rhie *et al.*, 2020). This work was done using Nextflow ( Di Tommaso *et al.*, 2017) DSL2 pipelines “sanger-tol/readmapping” ( Surana *et al.*, 2023a) and “sanger-tol/genomenote” ( Surana *et al.*, 2023b). The genome was analysed within the BlobToolKit environment ( Challis *et al.*, 2020) and BUSCO scores ( Manni *et al.*, 2021; Simão *et al.*, 2015) were calculated.

Table 3 contains a list of relevant software tool versions and sources.

**Table 3.** Software tools: versions and sources.

Software tool	Version	Source
BlobToolKit	4.1.7	https://github.com/blobtoolkit/blobtoolkit
BUSCO	5.3.2	https://gitlab.com/ezlab/busco
Hifiasm	0.16.1-r375	https://github.com/chhylp123/hifiasm
HiGlass	1.11.6	https://github.com/higlass/higlass
Merqury	MerquryFK	https://github.com/thegenemyers/MERQURY.FK
MitoHiFi	2	https://github.com/marcelauliano/MitoHiFi
PretextView	0.2	https://github.com/wtsi-hpag/PretextView
purge_dups	1.2.3	https://github.com/dfguan/purge_dups
sanger-tol/genomenote	v1.0	https://github.com/sanger-tol/genomenote
sanger-tol/readmapping	1.1.0	https://github.com/sanger-tol/readmapping/tree/1.1.0
YaHS	yahs-1.1.91eebc2	https://github.com/c-zhou/yahs

### Wellcome Sanger Institute – Legal and Governance

The materials that have contributed to this genome note have been supplied by a Darwin Tree of Life Partner. The submission of materials by a Darwin Tree of Life Partner is subject to the **‘Darwin Tree of Life Project Sampling Code of Practice’**, which can be found in full on the Darwin Tree of Life website here. By agreeing with and signing up to the Sampling Code of Practice, the Darwin Tree of Life Partner agrees they will meet the legal and ethical requirements and standards set out within this document in respect of all samples acquired for, and supplied to, the Darwin Tree of Life Project. 

Further, the Wellcome Sanger Institute employs a process whereby due diligence is carried out proportionate to the nature of the materials themselves, and the circumstances under which they have been/are to be collected and provided for use. The purpose of this is to address and mitigate any potential legal and/or ethical implications of receipt and use of the materials as part of the research project, and to ensure that in doing so we align with best practice wherever possible. The overarching areas of consideration are:

• Ethical review of provenance and sourcing of the material

• Legality of collection, transfer and use (national and international) 

Each transfer of samples is further undertaken according to a Research Collaboration Agreement or Material Transfer Agreement entered into by the Darwin Tree of Life Partner, Genome Research Limited (operating as the Wellcome Sanger Institute), and in some circumstances other Darwin Tree of Life collaborators.

## Data Availability

European Nucleotide Archive: *Lactarius evosmus* (fruity milkcap). Accession number PRJEB58661; https://identifiers.org/ena.embl/PRJEB58661. ( Wellcome Sanger Institute, 2023) The genome sequence is released openly for reuse. The *Lactarius evosmus* genome sequencing initiative is part of the Darwin Tree of Life (DToL) project. All raw sequence data and the assembly have been deposited in INSDC databases. The genome will be annotated using available RNA-Seq data and presented through the Ensembl pipeline at the European Bioinformatics Institute. Raw data and assembly accession identifiers are reported in Table 1.

## Author information

Members of the Royal Botanic Gardens Kew Genome Acquisition Lab are listed here: https://doi.org/10.5281/zenodo.4786680.

Members of the Darwin Tree of Life Barcoding collective are listed here: https://doi.org/10.5281/zenodo.4893703.

Members of the Wellcome Sanger Institute Tree of Life programme are listed here: https://doi.org/10.5281/zenodo.4783585.

Members of Wellcome Sanger Institute Scientific Operations: DNA Pipelines collective are listed here: https://doi.org/10.5281/zenodo.4790455.

Members of the Tree of Life Core Informatics collective are listed here: https://doi.org/10.5281/zenodo.5013541.

Members of the Darwin Tree of Life Consortium are listed here: https://doi.org/10.5281/zenodo.4783558.
