# Supplementary material for: The genome sequence of the Fruity Milkcap, Lactarius evosmus (Kühner & Romagn., 1954)
Source: Wellcome Open Res. 2023 Oct 18;8:471. [Version 1] doi: 10.12688/wellcomeopenres.19910.1 (PMC11157192; doi:10.12688/wellcomeopenres.19910.1)
Supplement: Peer review history

# Reviewer report: Reviewer response for version 1

**DOI:** 10.21956/wellcomeopenres.22046.r83758

**Published:** 2024-06-06

**Authors:** Bhagya C. Thimmappa

**Affiliations:** 1 Université de Montréal, Montréal, Canada

In the work titled “The Genome Sequence of the Fruity Milkcap, *Lactarius evosmus*,” the authors report nuclear and mitochondrial assembly for *Lactarius evosmus*, which was initially collected from Watlington Hill.

**Comments:**

- What are those missing Buscos? What does it tell us about ***Lactarius evosmus***?
- Is there a phylogenetic tree showing how ***Lactarius evosmus*** is placed with other closely related species? In other words, what are other closely related species?

Reported mitochondrial genome assembly could be a valuable resource for building better phylogenetic trees (provided mitochondrial genome assembly is available for other species).

Are sufficient details of methods and materials provided to allow replication by others?

Yes

Is the rationale for creating the dataset(s) clearly described?

Yes

Are the datasets clearly presented in a useable and accessible format?

Yes

Are the protocols appropriate and is the work technically sound?

Yes

Reviewer Expertise:

Fungal genomics and transcriptomics

I confirm that I have read this submission and believe that I have an appropriate level of expertise to confirm that it is of an acceptable scientific standard.

# Reviewer report: Reviewer response for version 1

**DOI:** 10.21956/wellcomeopenres.22046.r83759

**Published:** 2024-05-30

**Authors:** Nagarjun Vijay

**Affiliations:** 1 Indian Institute of Science Education and Research (IISER), Bhopal, MP, India

**Summary of the article**: Cullington et al., generate a high quality genome assembly of the Fruity Milkcap, *Lactarius evosmus*. The genome is assembled using 326x coverage PacBio along with Hi-C data. Standard genome assembly metrics such as the BlobToolKit Snailplot, GC-coverage plot, cumulative sequence plot, Hi-C contact map are provided.

 Major comments:

 1) The rationale for creating the datasets appears to be "The genome of *Lactarius evosmus* was sequenced as part of the Darwin Tree of Life Project, a collaborative effort to sequence all named eukaryotic species in the Atlantic Archipelago of Britain and Ireland."

 Please provide some details regarding what are the actual questions of interest, how this genome fits into the broader scheme of things, such as other genomes being sequenced etc.

 2) Some context for the genome assembly metrics and how it compared to either other species with similar genomes or to the best genome assembly possible would be important. 

 Unfortunately, no effort is made to evaluate the "validity" of the genome assembly. Have you considered using some post-assembly validation tools like Reaper?

 3) Comparing the assembly structure with the genome of other closely related species should also provide some idea of how well the assembly has performed

Are sufficient details of methods and materials provided to allow replication by others?

Yes

Is the rationale for creating the dataset(s) clearly described?

Partly

Are the datasets clearly presented in a useable and accessible format?

Yes

Are the protocols appropriate and is the work technically sound?

Yes

Reviewer Expertise:

Genome assembly and bioinformatics

I confirm that I have read this submission and believe that I have an appropriate level of expertise to confirm that it is of an acceptable scientific standard.
